# Multifunctional Templates for Minimized Osteotomy, Implantation, and Palatal Distraction with a Mini-Screw-Assisted Expander in Schizodontism and Maxillary Deficit

**DOI:** 10.1155/2020/8816813

**Published:** 2020-09-09

**Authors:** Manfred Nilius, Katrin Hess, Dominik Haim, Bernhard Weiland, Guenter Lauer

**Affiliations:** ^1^Niliusklinik, Londoner Bogen 6, D-44269 Dortmund, Germany; ^2^Department of Oral and Maxillofacial Surgery, University Hospital “Carl Gustav Carus”, Technische Universität Dresden, Fetscherstr. 74, D-01307 Dresden, Germany

## Abstract

**Purpose:**

Schizodontism is complete separation of a dental germ. It results in a twin tooth and supernumerary teeth. The treatment of transverse constriction in combination with supernumerary dental germs and impacted central incisors can pose a challenge, especially in young patients, when the number of permanent teeth is not adequate to ensure secure anchorage. The use of navigation templates based on three-dimensional X-ray images allows for precise insertion of temporary mini-implants for the acquisition of palatal distractors. In addition, templates allow for minimally invasive biopsies and osteotomies.

**Methods:**

The treatment of schizodontism, dentitio tarda, and transverse constriction is to be assessed as an interdisciplinary method by using mini-screw-assisted devices. Minimized osteotomy of impacted supernumerary teeth or dental implantation can be carried out in a one-step-procedure based on digital preplanning and prefabrication of orthodontic devices.

**Results:**

Multifunctional templates allow for early planning, preoperative fabrication, and intraoral fixation of orthodontic appliances. In the case of an adolescent patient, a sustainable, interdisciplinary treatment concept could be demonstrated that shows age-appropriate gnathological development and stable growth conditions over a follow-up period of 10 years.

**Conclusion:**

One can likely assume that multifunctional templates allow for minimally invasive one-step surgeries as an interdisciplinary tool between orofacial surgery and modern orthodontics.

## 1. Introduction

Schizodontism, germination, and twinning are uncommon developmental anomalies of the hard dental tissue. These aberrations are manifested either as anomalous teeth or supernumerary teeth [1, 2]. The prevalence rate is variable in individual reports, and the overall prevalence appears to be approximately 0.5% in the deciduous teeth and 0.1% in the permanent dentition. It is more prevalent in the anterior maxillary region affecting incisors and canines. Bilateral cases are seen less frequently, with a prevalence of 0.02% in both dentitions [3]. Cone beam computed tomography (CB-CT) is a three-dimensional (3D) imaging technique that allows proper localization of supernumerary teeth, measurement of palatal constriction, and treatment planning prior to orthodontic or surgical intervention [2]. The diagnostic workflow and treatment concept by using a mini-screw-assisted device for guided osteotomy, palatal distraction, and orthodontic treatment will be presented and critically discussed.

Schizodontism, intermaxillary incongruences, and transverse constriction will be discovered early in the context of child or adolescent medical check-ups. This means that patients are given dental and orthodontic treatment at an early stage. A precise and carefully considered strategy of treatment is therefore important. Intervention should be as gentle as possible to reduce comorbidities. In addition, predictability and sustainability should be mandatory. Orofacial backward-planning enables surgical interventions to be carried out in a minimally invasive manner, bundled with orthodontic treatment, and could be—if necessary—reduced to a minimum.

## 2. Case Presentation

A 12-year-old girl visited the dental clinic because of delayed tooth eruption and deciduous tooth persistence. The first tooth breakthrough for deciduous incisors was observed at the age of 14 months. The family dental history was inconspicuous. Pediatric check-ups were performed regularly and a percentile conformal normal growth was found.

## 3. Clinical Findings

The clinical findings showed a brachyfacial facial type corresponding to the chronological age. Intraoral eruption of the six-year molars all lower incisors. In addition, the lateral upper incisors were broken through, rotated, and had a gap to the canines. There was persistence of all deciduous molars and deciduous canines. Owing to the absence of the anterior tooth breakthrough, a functional switching gap resulted between the central maxillary incisors. The anterior and posterior arch width of the maxilla was reduced.

## 4. Radiological Findings

**Figure 1:**
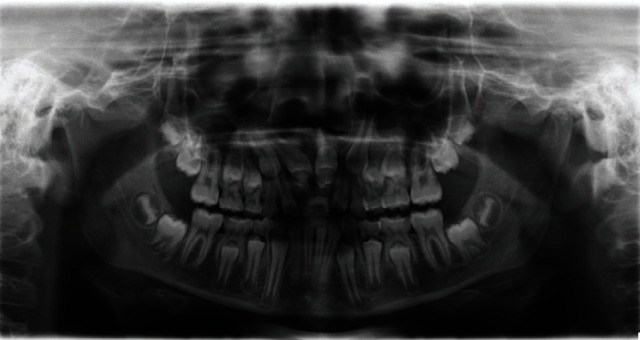
Orthopantomogram (OPG) at the age of eleven: the retention and the double formation of the central incisors can be seen.

**Figure 2:**
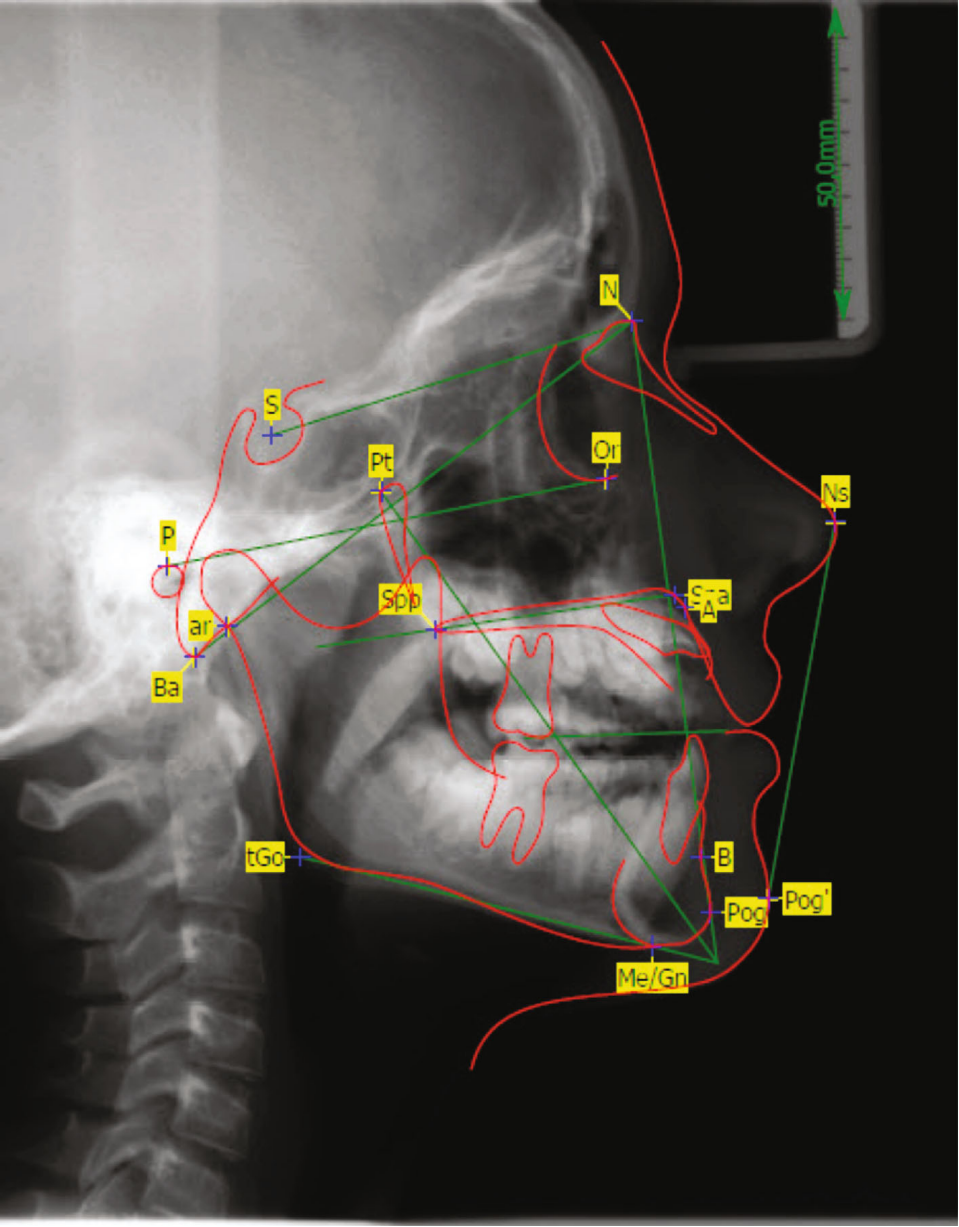
Lateral cephalometric radiographs at the age of eleven; for abbreviations, see also Table 1.

**Figure 3:**
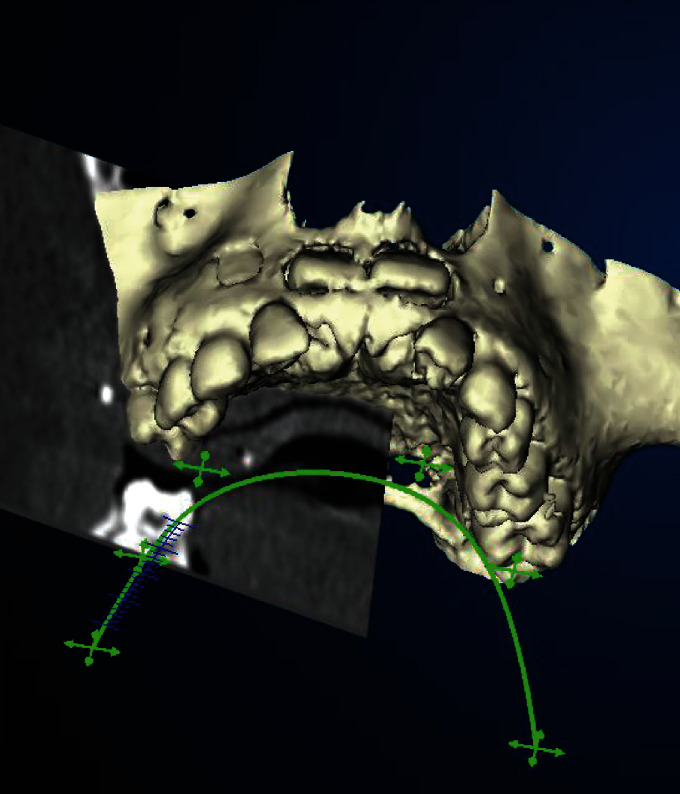
Digital volume tomography; CB-CT (3D-Kavo Exam) in the caudolaterocranial view with projected upper jaw line (green) and transversal section plane.

Two supernumerary dorsocaudopalatal displaced microforms of central incisors (schizodonts: 11b, 21b; Figures 1–3) and nasocranial-positioned normal forms of incisors (11, 21) were seen on radiographics (orthopantomography (OPG); Orthophos XG, Sirona Dental Systems, Bensheim, Germany), digital volume tomography (CB-CT; KaVo 3D eXam ConeBeam XG; KaVo Dental GmbH, Biberach/Riss, Germany), and lateral cephalometric view (Orthophos XG, Sirona Dental Systems, Bensheim, Germany). The software used for CB-CT was eXam-VisionQ®, Version 1.9.3.13 (Kaltenbach & Vogt Dental GmbH, Biberach/Riss, Germany). The X-ray images corresponded to the status of a mixed dentition at the end of the first dentition with persistence of 55, 54, 53, 63, 64, and 65 and retention of all permanent tooth germs, with narrow germination of 13 and 23.

## 5. Diagnosis

- Schizodontism of 11 and 21 with palatal dislocated schizodonts (microforms) [4, 5]
- Persistence of deciduous teeth [4]
- Transverse constriction of the upper jaw; narrow germination of 13 and 23

## 6. Therapy

- Exposure and orthodontic alignment of the subnasal displaced teeth 11 and 21
- Minimally invasive removal of the palatal displaced schizodonts 11b and 21b
- Transverse development of the maxilla by implant-supported palatal distraction

## 7. Planning: The Template as a Multifunctional Tool

**Figure 4:**
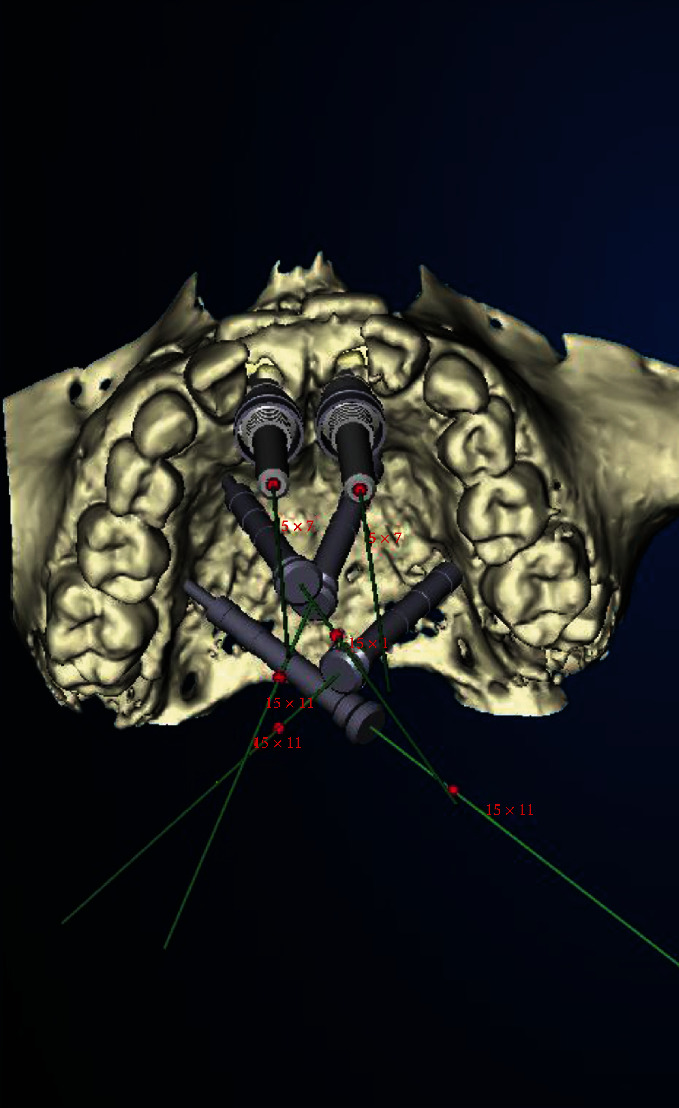
NobelGuide® planning for guided biopsy and palatal anchoring of 4 diverging anchor pins (Nobel Biocare Services®, Klothen, Switzerland).

**Figure 5:**
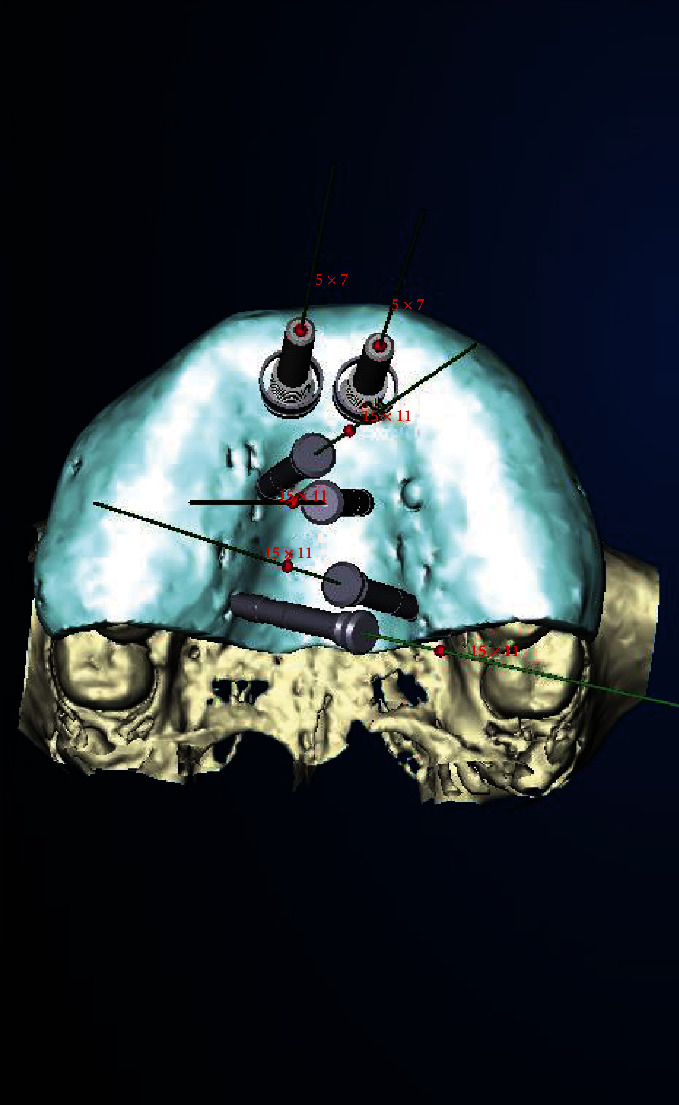
Modified NobelGuide® template for guided biopsy.

**Figure 6:**
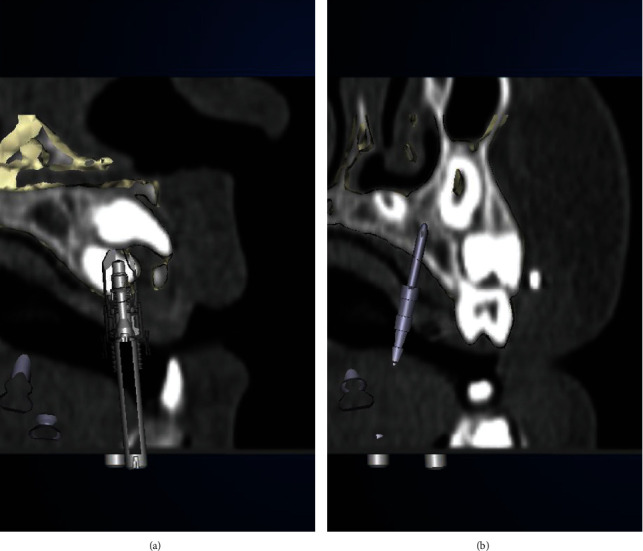
Transdental implant planning for guided biopsy of schizodonts (a) and palatal distraction using anchor pins in region 65 (b).

A model and CB-CT analyses were used to design a special NobelGuide template [6] with several different functions (Figures 4–6):

- Guided insertion of mini-implants with immediate loading: as an anchorage-tool, mini-implants were planned, placed, and loaded immediately with a palatal distractor
- Minimally invasive biopsy: templates were used for the targeted, minimally invasive removal of palatal displaced schizodonts. The horizontally displaced incisors were provided with vestibular brackets and aligned by traction
- Implant-guided palatal distraction (IGPD): to obtain space for the horizontally displaced incisors (11, 21), transverse distraction was necessary [7]. To align the exposed teeth, the distractor was extended anteriorly to hold the elastics or wire ligatures

## 8. Prefabrication of the Individual Implant-Guided Palatal Distractor (IGPD)

**Figure 7:**
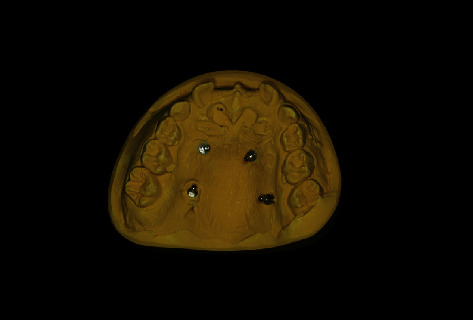
Planning model with implant analogues to fix the Implant-Guided Palatal Distractor (IGPD).

**Figure 8:**
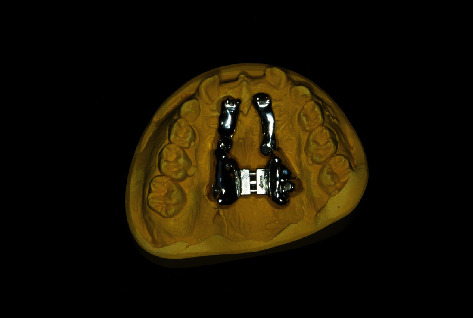
Planning model with fixed IGPD.

A special model with implant analogues (Nobel Biocare Services®, Klothen, Switzerland) was prefabricated (Figure 7) by using the template. Then, 8° conical abutments were placed on each implant-analogue. A mesiostructure with 2° milled bars and two anterior extensions was added to each extension to hold the ligature wire. We rigidly fixed this device with frictional fit over the bars and began to activate transverse expanding by use of a Memory Palatal Split Screw (No. 167 M1529, Forestadent® Pforzheim; Germany) (Figure 8). Owing to divergence of the mini-implants, the superstructure had to be fabricated in the following 3 parts: part 1—8° cone construction; part 2—attached bar in two parts; and part 3—connector with a memory screw.

## 9. Surgical Procedure

**Figure 9:**
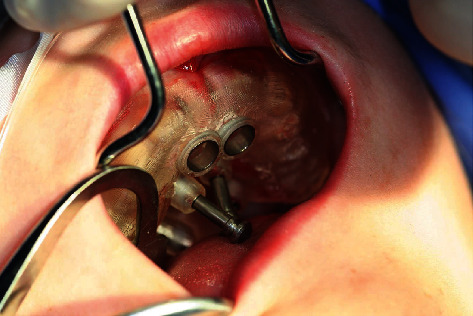
NobelGuide® template in situ after insertion of 2 anchor pins.

**Figure 10:**
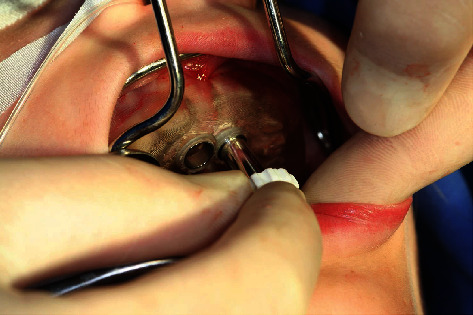
Guided-Tissue-Punch® for mucosal punching.

**Figure 11:**
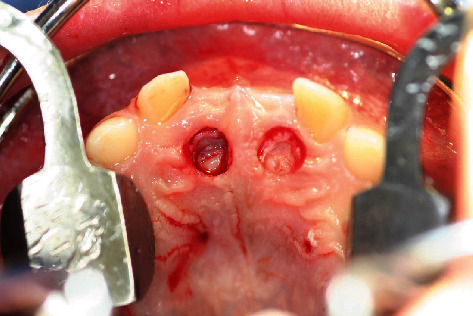
Coronal parts of the schizodonts after mucosal punching.

**Figure 12:**
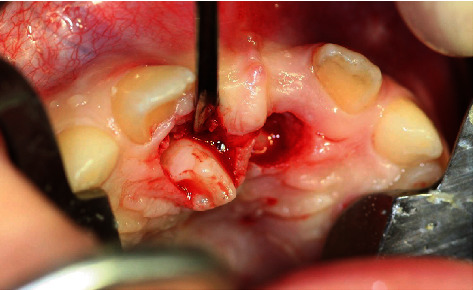
Coronal parts of the schizodonts after mucosal punching (detail).

**Figure 13:**
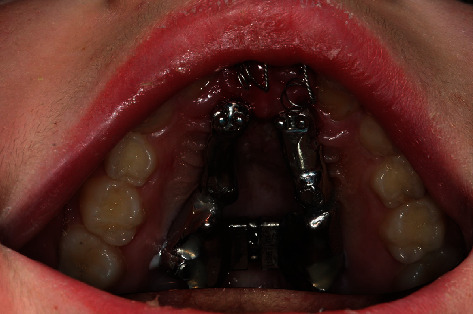
Fixed IGPD (not enabled).

**Figure 14:**
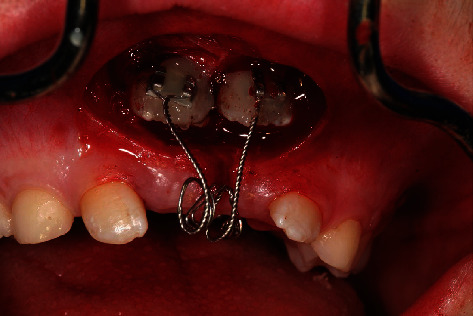
Exposure of the displaced teeth 11 and 21 with brackets and wire ligature.

At first, the NobelGuide® template was positioned on the maxillary teeth. For osseous fixation, four diverging anchor pins (Nobel Biocare Services®, Zurich, Switzerland; diameter: 1.5 mm) were inserted from the palatal side (Figure 9). Guided punching of the mucosa (Soft Tissue Punch®, Nobel Biocare Services®, Zurich, Switzerland; diameter: 5.2 mm) was added (Figures 10 and 11). Osteotomy of the palatal compacta uncovered the schizodonts for extraction (Figure 12). The harvested gingival punch was replaced palatal to the mucosa. We removed the template and anchor pins (1.5 mm x 10 mm) and replaced them with primary stable immediate implants (2.8 mm x 13.0 mm) by using the implant holes. The cone connectors of the mesiostructure were cemented precisely to the patrixes of the immediate implants. The anterior branches were located at the level of the palatal gingiva of 12 and 22. Then, the distractor was attached to the mesiostructure. The distal ligature ends were fixed to the anterior branches of the distractor (Figures 8 and 13). The two permanent anterior teeth were exposed from the vestibular side. Brackets were fixed adhesively using the acid etching technique and 11 and 21 were attached by ligatures (Figure 14) to the branches of the mesiostructure.

## 10. Postoperative Course

**Figure 15:**
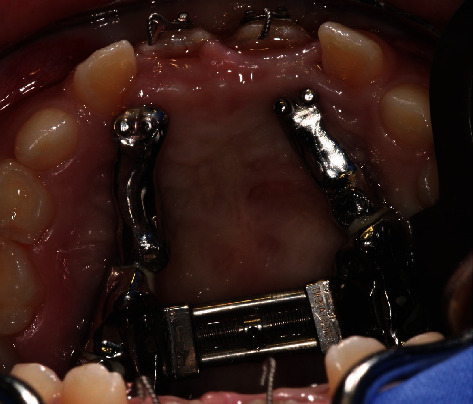
Fixed IGPD (18 days postoperative, enabled).

**Figure 16:**
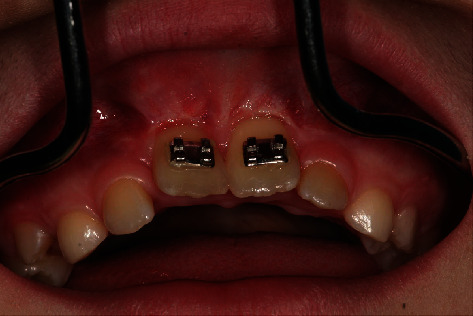
Follow-up 6 months postoperative.

Over a period of 18 days, discontinuous palatal distraction of the posterior dental arch (7 mm) was performed. Finally, the transverse screw was blocked to remain in position. The follow-up after 12 weeks showed a stable gap widening along the entire Raphe median plane up to the anterior region (Figure 15). The planned lowering and ventral alignment of the central incisors also became evident (Figure 16). The orthodontic treatment was continued over a period of 4 years (brackets: In-Ovation C Base 0.018″ Roth Slot and In-Ovation R 0.018^″^Roth Slot; wire: Sentalloy Accu-med. blue 0.16^″^, Dentsply Sirona, Bensheim, Germany; wire sequence: 0.012^″^ × 0.014^″^, 0.016^″^ × 0.022^″^ Sentalloy blue, and finally 0.16^″^ × 0.22^″^ stainless steel, “Ideal-Arch”; Snap-Fit convertible triple tube Roth, Dentsply Sirona, Bensheim, Germany).

**Figure 17:**
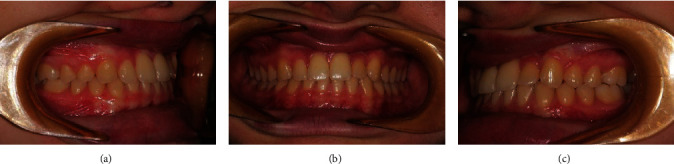
(a, b, c) Follow-up 48 months postoperative (end of treatment).

**Figure 18:**
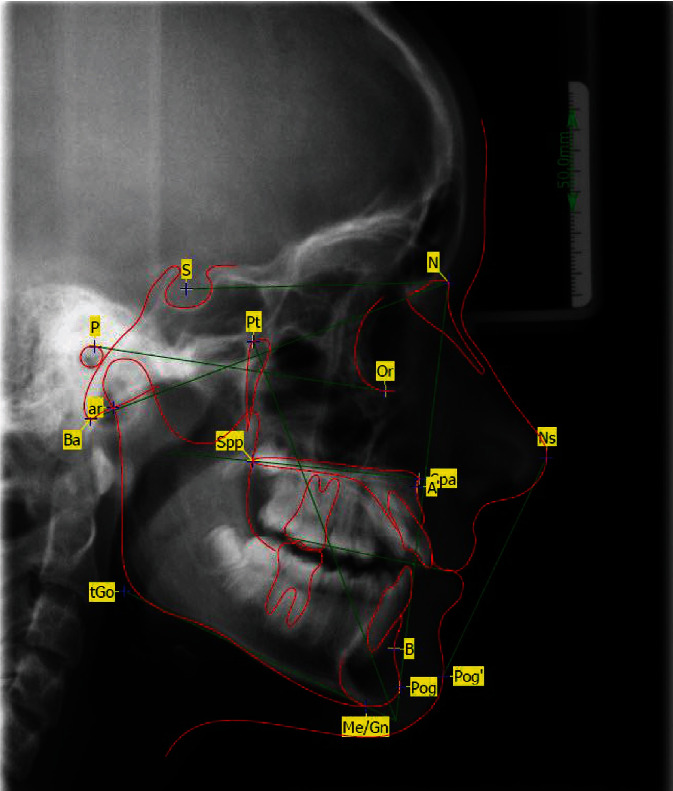
Lateral cephalometric radiograph with evaluation 12 months postoperative; for abbreviations, see also Table 1.

**Figure 19:**
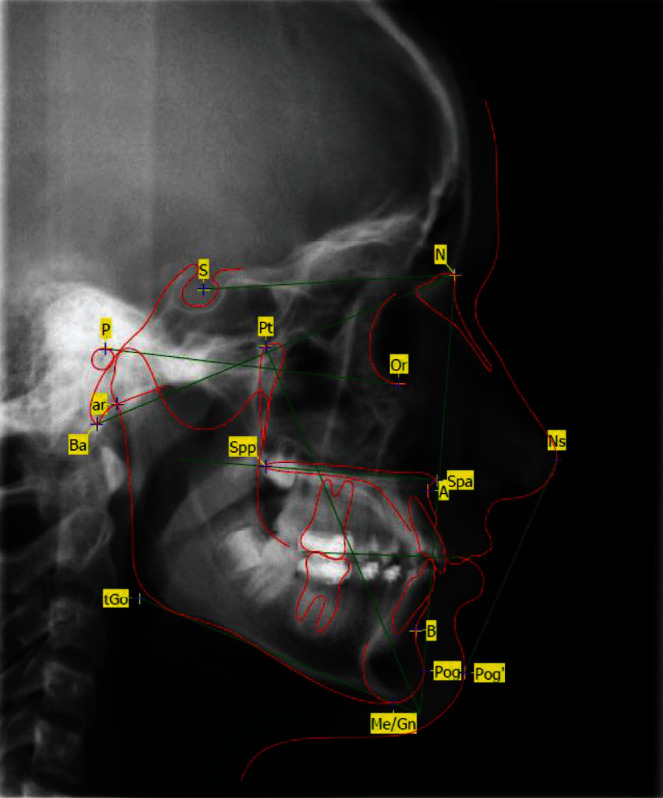
Lateral cephalometric radiograph with evaluation 48 months postoperative (end of treatment); for abbreviations, see also Table 1.

It resulted in complete alignment of the central anterior teeth (Figure 17). The vitality test of all permanent teeth after breakthrough was positive. The 6-year radiological follow-up showed that the central incisors were in the correct position according to the standards [8]. The initially protruded front teeth to OK1 SN: 138° (Figure 2) could be retruded to an inclination of OK1 to SN: 108° (Figure 18). A normal interincisal angle of 133° was achieved by compensatory protrusion of the lower incisors. The previously retruded lip profile was compensated. The lateral cephalometric radiographs showed development from the brachyfacial- to the mesiofacial-type during the treatment (Figures 2, 18, 17, and 19) [9]. For abbreviations, see Table 1.

## 11. Discussion

### 11.1. The Navigated Implant and Biopsy Planning

The NobelGuide planning is easily linkable with intraoral scanners. This technology has registered a constantly increasing use in many fields of dentistry, such as restorative dentistry, prosthodontics, orthodontics, and implantology. It allows for a completely digital workflow, from impression to final framework, with clinical reliability (precision of the virtual occlusal record) [10] and good patient feedback [11].

In conventional NobelGuide planning, the anchor pins are used to fix the position of the template in the edentulous jaw and the implants are placed in the dental arch in a planned and navigated manner. In our modified case, the IGPD fixed the template to the deciduous teeth; they serve as the dental support [6]. The actual implant planning involves guided biopsy of the palatal schizodonts. At best, it is planned with the largest diameter (*x* > 5 mm). The anchor pins serve to position and fix the template precisely during the biopsy. In addition, the position of the anchor pins can be used as a pilot hole for the mini-implants and the primary stable insertion (1.5 pilot hole; impl. diameter, 2.8 mm). Owing to the exact drilling in the palatal D1-D2 maxilla bone, a high insertion torque of *x* > 35 N cm can be achieved [6, 12]. This allows for immediate loading of the implants. Furthermore, single-stage insertion of the IGPD can be implemented with early loading. This makes the performance of intraoperative impressions and a second surgical intervention unnecessary, which is particularly important in younger or anxious patients. The CB-CT recording technique offers the best accuracy for the described navigation-supported intervention in the first dentition, because there are usually no artifacts due to metallic restorations [6]. Despite an inaccuracy of 0.3 mm due to the system, this technique is also successful in CB-CT-assisted surgery; however, there is higher radiation intensity. In cases of palatal implant insertion with divergent implant axes, it is essential to pay attention to the roots of the permanent teeth. Algorithms for hard tissue usually allow a good differentiation of the teeth from the bone substance in the CB-CT analysis [12]. This allows IGPDs to be planned and clinically implemented with a high degree of accuracy [6, 7]. The alternative of a mere implant at insertion along the raphe median plane would be associated with a lower morbidity with respect to the tooth germs, but it does not allow for the use of mini-implants for palatal extension. Systems that are only dental supported (e.g., the hyrax screw) often lead to tooth displacement or accelerated resorption of deciduous teeth without any real transverse increase.

### 11.2. Tooth-Borne Apparatus versus Bone-Borne Apparatus

Owing to the generally delayed tooth penetration, our patient did not have enough permanent teeth available for dental anchorage of the palatal expansion appliance and the looping of the first incisors. Even after transverse expansion with a conventional removable expansion plate and pulling of the attached upper first incisors against it, the plate would not have provided support due to the beginning of tooth change in the second mixed dentition. Mini-screw-assisted palatal expanders are supported with titanium [13], stainless steel [14], or orthodontic miniscrews, like the used bone-borne apparatus (IGPD). In addition, anchoring by implants offers the advantage that neither the setting of the incisors nor the palatal expansion is dependent on the compliance of the young patient. Owing to stable connection of the IGPD with the upper jaw, the forces can be applied in a targeted, dosed manner and 24 hours a day. Owing to the 3D representation on the CB-CT, the IGPD could be constructed in such a way that an optimal pulling direction for the upper first incisors could be realized. However, a possibly necessary change in the pulling direction would have been difficult [15]. The inserted palatal implants transfer the forces of the expansion screw directly to the upper jaw, thus avoiding the load and potential overload of the palatal expansion appliance on the anchor teeth. This eliminates the typical risks of forced transverse expansion (bite opening due to buccal tilting of the first molars and root resorption of the anchor teeth). The lowering of the nasal floor leads to an improvement in the nasal passage (nasal airflow) and thus often leads to a spontaneous change from oral to nasal breathing [15]. If the patient's midface hypoplasia would have been more pronounced, the IGPD could have also served as an anchor for a Delaire mask. In the present case, however, the case was finished with moderate dental compensation for a slightly mesial bite (Figure 19), even if the jaws—see lateral view (Figure 18)—were not in maximum intercuspation during the course of the treatment. The bone-borne apparatus requires good oral hygiene in the patient. Otherwise, peri-implant inflammation could occur. Implant loosening is a rare complication with orthodontic mini-implants. Miniscrews inserted in midpalatal locations have shown a failure rate of 1.3% [16]. Owing to soldering of the IGPD construction, explantation of all implants had to be performed simultaneously. However, in the present case, this was not a disadvantage, because the setting of the incisors coincided approximately with the end of the retention time for the transverse expansion. The IGPD was relatively flat; thus, speech was hardly affected. Moderate impressions on the back of the tongue occurred only in the initial period after insertion of the appliance.

In summary, IGPD or other mini-screw-supported devices present good clinical reliability [17] and excellent mechanical properties even with small diameters [18]. The presented patient case shows that CB-CT-based planning and the preoperative appliance (customized distractor) improved the predictability of the surgical result, and it achieved a minimally invasive guided biopsy and a shortened duration of surgery. In addition, the subsequent orthodontic therapy could be conducted effectively and without any anchorage loss due to the inserted mini-implants. However, the mini-screw-supported technique is more invasive and presents a minimal risk of mini-screw fracture if compared with the conventional banded technique.

Supernumerary teeth often cause palatal constriction and are a therapeutic challenge for orthodontists and oral surgeons. Therefore, interdisciplinary therapeutic concepts are needed for the benefit of the patient.

Treatment planning depends on various factors, such as the time of diagnosis, the age of the patient, the position of the supernumerary tooth, and possible complications. This case report presents only one possible treatment option for a multidisciplinary approach in the supernumerary permanent dentition.

## Figures and Tables

**Table 1:** Orthodontic terms.

Variable	Description
*Skeletal analyses*	
SNA	SNA angle
SNB	SNB angle
ANB	ANB angle
A-NPog	Skeletal profile convexity
WITS	WITS appraisal
A-NPog	Distance from A to the facial plane
B-NP	Distance from B to the facial plane
NBaPtG	Facial axis angle
NPogPOr	Facial plane angle
MeGoPOr	Mandibular plane angle
ML-NSL	Inclination of the mandibular plane to the skull base
S-Go/N-Me %	Ratio between anterior and posterior face height
arGoMe	Gonial angle
Sum	Björk sum angle
SpaXiPm	Lower face height angle
ML-NL	Angle between the maxilla and the mandibular plane
SpaSppPOr	Palatal plane to FH plane (Frankfort horizontal)
NL-NSL	Inclination of the maxilla to the skull base
OcP-NL	Angle between occlusal plane and maxilla
*Dental analysis*	
II	Interincisal angle
OK1 to SN	Inclination of the upper central to sella-nasion-line
OK1 to A-Pog	Distance from the upper central incisor to A-Pog-line
OK1 to A-Pog angle	Angle between the upper central incisor and A-Pog-line
UK1 to A-Pog	Distance from the lower central incisor to A-Pog-line
UK1 to A-Pog angle	Angle between the lower central incisor and A-Pog-line
UK1 to Go-Me	Angle between the lower central incisor and mandibular plane
OK6 to PTV	Distance from the upper first molar to PTV
OK1 to occlusal plane	Distance from the upper central incisor to the occlusal plane
*Profile*	
Lower lip to E-plane	Distance from the lower lip to the aesthetic line
Gl′-Sn/Sn-Me′	Angle between glabella-subnasale line and subnasale-menton′ line
Sn-Sto/Sto-Me′	Angle between subnasale-stomion line and stomion-menton′ line
Sn-Li/Li-Me′	Angle between subnasale-lower lip line and lower lip-menton′ line
GI′-Sn-Pog′	Profile angle
Cotg-Sn-Ls	Nasolabial angle
